# ACCURACY OF CT NUMBERS OBTAINED BY DIRA AND MONOENERGETIC PLUS ALGORITHMS IN DUAL-ENERGY COMPUTED TOMOGRAPHY

**DOI:** 10.1093/rpd/ncab108

**Published:** 2021-07-16

**Authors:** Maria Magnusson, Michael Sandborg, Gudrun Alm Carlsson, Lilian Henriksson, Åsa Carlsson Tedgren, Alexandr Malusek

**Affiliations:** Department of Electrical Engineering; Linköping University, SE-581 83, Linköping, Sweden; Department of Health, Medicine and Caring Sciences; Linköping University, SE-581 83, Linköping, Sweden; Center for Medical Image Science and Visualization (CMIV); Linköping University, SE-581 85, Linköping, Sweden; Department of Health, Medicine and Caring Sciences; Linköping University, SE-581 83, Linköping, Sweden; Center for Medical Image Science and Visualization (CMIV); Linköping University, SE-581 85, Linköping, Sweden; Department of Health, Medicine and Caring Sciences; Linköping University, SE-581 83, Linköping, Sweden; Center for Medical Image Science and Visualization (CMIV); Linköping University, SE-581 85, Linköping, Sweden; Department of Health, Medicine and Caring Sciences; Linköping University, SE-581 83, Linköping, Sweden; Center for Medical Image Science and Visualization (CMIV); Linköping University, SE-581 85, Linköping, Sweden; Department of Health, Medicine and Caring Sciences; Linköping University, SE-581 83, Linköping, Sweden; Center for Medical Image Science and Visualization (CMIV); Linköping University, SE-581 85, Linköping, Sweden; Department of Medical Radiation Physics and Nuclear Medicine; Karolinska University Hospital, SE-171 77, Stockholm, Sweden; Department of Health, Medicine and Caring Sciences; Linköping University, SE-581 83, Linköping, Sweden; Center for Medical Image Science and Visualization (CMIV); Linköping University, SE-581 85, Linköping, Sweden

## Abstract

Dual-energy computed tomography (CT) can be used in radiotherapy treatment planning for the calculation of absorbed dose distributions. The aim of this work is to evaluate whether there is room for improvement in the accuracy of the Monoenergetic Plus algorithm by Siemens Healthineers. A Siemens SOMATOM Force scanner was used to scan a cylindrical polymethyl methacrylate phantom with four rod-inserts made of different materials. Images were reconstructed using ADMIRE and processed with Monoenergetic Plus. The resulting CT numbers were compared with tabulated values and values simulated by the proof-of-a-concept algorithm DIRA developed by the authors. Both the Monoenergetic Plus and DIRA algorithms performed well; the accuracy of attenuation coefficients was better than about ±1% at the energy of 70 keV. Compared with DIRA, the worse performance of Monoenergetic Plus was caused by its (i) two-material decomposition to iodine and water and (ii) imperfect suppression of the beam hardening artifact in ADMIRE.

## INTRODUCTION

The ability of computed tomography (CT) to produce information about photon attenuation of imaged objects is widely used in medical diagnostics and radiotherapy planning. The latter requires accurate attenuation data to calculate doses delivered during radiotherapy treatments, particularly proton therapy and low-energy brachytherapy. A typical workflow is that electron densities are determined first^(1)^, and from those, voxel-specific cross sections are determined by making assumptions about the material composition of individual tissues^(2)^. Attenuation data provided by single-energy CT suffer from beam hardening artifacts^(3)^. Dual-energy CT, which scans the patient using two different X-ray tube voltages, can suppress the beam hardening artifacts by performing material decomposition. In the Alvarez–Macovski method^(4)^, the decomposition is performed on the projection data. This method requires geometrically consistent rays from both low- and high-energy scans, and therefore its use is currently limited to (i) the dual-layer DECT technique^(5)^ available in the IQon Spectral CT (Philips Healthcare) and (ii) the fast kV switching technique^(6)^ available for instance in the Revolution CT (GE Healthcare). In the case of the dual-source DECT acquisition technique^(7)^, the material decomposition is performed on reconstructed images; examples are the Monoenergetic Plus algorithm by Siemens Healthineers^(8)^ and the proof-of-concept algorithm DIRA developed by the authors^(9)^.

In Monoenergetic Plus, which has been used for radiotherapy planning^(10)^, images reconstructed from low- and high-tube voltage projections are decomposed via the two-material decomposition method to water and iodine. The resulting mass fractions are then used to calculate the virtual monoenergetic images as in DIRA. To suppress image noise, the algorithm utilizes low spatial frequencies from the low energy image and high spatial frequencies from the high energy image; low energy levels generally provide better contrast but suffer from higher noise. By having access to virtual monoenergetic images at different energy levels, the most suitable level can be chosen for the current clinical task.

In DIRA, projections are first reconstructed using filtered back-projection. Then, in the simple version, the images are segmented into bones and soft tissues. Bones are decomposed to compact bone and bone marrow using a two-material decomposition. Soft tissues are decomposed to water, protein and lipid using a three-material decomposition. Obtained base material mass fractions are used to construct a model of the patient. Simulated monoenergetic and polyenergetic projections of the model are then used to calculate corrections that convert polyenergetic projections to monoenergetic ones, which are not affected by the beam hardening artifact. These steps are iterated until no further improvement in the accuracy of reconstructed data is observed, typically 10 times. Finally, virtual monoenergetic images are calculated from reconstructed mass fractions of base materials.

This paper aims to evaluate whether there is room for improvement of the Monoenergetic Plus algorithm regarding the accuracy of reconstructed CT numbers. First, by comparing the measured CT numbers with tabulated values derived from the EPDL97 library. This comparison represents a theoretical limit, which may be difficult to reach in practice. Second, by comparing the measured values with those obtained via computer simulations via a proof-of-concept image reconstruction algorithm DIRA.

## MATERIALS AND METHODS

Measured CT numbers obtained using the Monoenergetic Plus algorithm were compared with CT numbers calculated using (i) elemental composition of the materials, (ii) two-material decomposition to the (water and iodine) doublet and (iii) DIRA.

### Measured CT numbers

**Figure 1:**
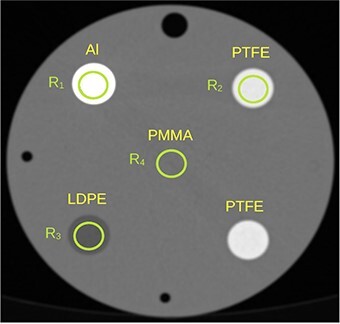
Rod inserts and ROIs, R_1_,…,R_4_, in the PMMA phantom. The ROIs were smaller than the rods to avoid edge effects.

A Siemens SOMATOM Force scanner (Siemens Healthineers) was used to scan a cylindrical polymethyl methacrylate (PMMA) phantom of the diameter of 160 mm with four rod inserts of diameter 20 mm made of aluminium, polytetrafluoroethylene (PTFE, Teflon), and low-density polyethylene (LDPE), see Figure 1. Peripheral holes for a pencil ion chamber were filled with air.

A clinical-like, helical, dual-energy protocol with 80 and 150 kV with an added Sn filter (denoted as Sn150kV) was used. Images were reconstructed with the ADMIRE algorithm (Qr36d kernel, strength 2) in the Siemens’ syngo.via 5.1 software both without and with the optional iterative beam hardening correction (iBHC) applied. Virtual monoenergetic images at 40, 60, 70, 80, 100, 120, 140, 160 and 180 keV were obtained from the reconstructed images using the Monoenergetic Plus (version VB30) application profile of the Siemens’ CT Dual-Energy software. Averages of CT numbers in selected regions of interest (ROIs) were obtained using the ImageJ software (https://imagej.nih.gov/ij/), other data analysis was done using MATLAB (https://mathworks.com/) and R (https://www.r-project.org/).

### CT numbers calculated using elemental composition and tabulated values

The CT number, $H$, of each phantom material was calculated as

(1) \begin{equation*} H=1000\cdot \left(\frac{\mu }{\mu_w}-1\right), \end{equation*}

where $\mu$ and ${\mu}_w$ are the linear attenuation coefficients (LAC) of the material and water, respectively, at specific photon energy. These values were calculated using the independent atom approximation from the known elemental composition of the materials as

(2) \begin{equation*} \mu =\rho{\sum}_i{w}_i{\mu}_{\mathrm{m},i}, \end{equation*}

where $\rho$ is the measured mass density of the material, and ${w}_i$ and ${\mu}_{\mathrm{m},i}$ are the mass fraction and the mass attenuation coefficient, respectively, of the *i*:th element in the mixture. Mass attenuation coefficients were derived from the EPDL97 library^(11)^. Elemental mass fractions were derived from molecular formulas.

### CT numbers predicted by the (water and iodine) material base

The CT number, $H$, predicted by the (water and iodine) material base was calculated using Equation (1) from the LAC $\mu (E)$ calculated as

(3) \begin{equation*} \mu (E)={\rho}_w{\mu}_{\mathrm{m},\mathrm{w}}(E)+{\rho}_I{\mu}_{\mathrm{m},\mathrm{I}}(E), \end{equation*}

where ${\mu}_{\mathrm{m},\mathrm{w}}(E)$ and ${\mu}_{\mathrm{m},\mathrm{I}}(E)$ are tabulated mass attenuation coefficients for water and iodine, respectively, at the photon energy $E$. The partial mass densities ${\rho}_w$ and ${\rho}_I$ for water and iodine, respectively, were calculated from a system of two equations derived from Equation (3) by setting $E$ to ${E}_1=50$ keV and ${E}_2=93$ keV in the first and second equation, respectively; $\mu ({E}_1)$ and $\mu ({E}_2)$ were the measured LAC. This system requires that the linear superposition of the tabulated values equals the LAC of the material at two photon energies ${E}_1$ and ${E}_2$, whose values corresponded to effective energies of the 80 kV and Sn150kV spectra.

### CT numbers calculated using DIRA

Dimensions and material composition of the mathematical phantom resembled those of the real phantom; small holes filled with air were not simulated. Dimensions, X-ray tube filters, bowtie-filter and detector array configuration of the simulation geometry resembled the ones of the SOMATOM Force scanner. The bowtie-filter model was developed by the authors according to specifications provided by Siemens under an NDA. Axial X-ray projections were calculated using the Drasim code^(12)^ for 80 kV and Sn150kV. Quantum noise and patient table were not simulated. The projection data were then processed with DIRA, which used a two-material decomposition to the bone and bone marrow doublet and a three-material decomposition to the lipid, protein and water triplet. Virtual monoenergetic images were calculated from the computed material mass fractions and mass attenuation coefficients. Material data used in DIRA and Drasim were derived from the EPDL97 data library^(11)^.

## RESULTS

We recall that images reconstructed with ADMIRE at 80 kV and Sn150kV as input to Monoenergetic Plus, and thus artifacts introduced during the reconstruction process at this stage were further propagated to the virtual monoenergetic images.

### Images produced by ADMIRE

**Figure 2:**
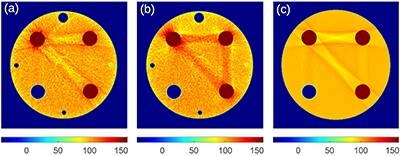
Images at 80 kV computed with (**a**) ADMIRE without iBHC (**b**) ADMIRE with iBHC and (**c**) DIRA at iteration 0 consisting of FBP with water beam hardening correction. ADMIRE processed measured data, DIRA processed simulated data. For DIRA: small holes, noise, kernel and patient table were not simulated. The range of CT numbers is adjusted to highlight beam hardening.

**Figure 3:**
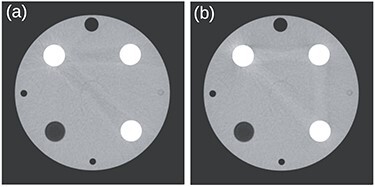
Images reconstructed with ADMIRE strength two at 80 kV without (**a**) and with (**b**) iBHC. Images are displayed with a standard window setting for an abdominal soft tissue exam (level of 50 HU, width of 400 HU).

**Figure 4:**
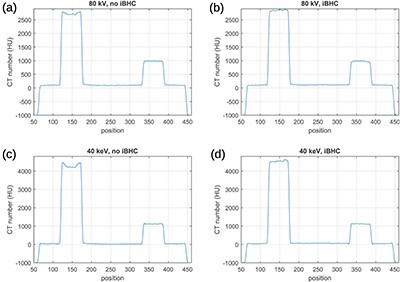
Profiles of images reconstructed with ADMIRE at 80 kV (**a**) without and (**b**) with iBHC and images reconstructed with Monoenergetic Plus at 40 keV (**c**) without and (**d**) with iBHC. The profiles were taken at a horizontal line passing through the aluminium and Teflon rods in Figures 2 and 5.

Figure 2 compares images reconstructed via ADMIRE at 80 kV without and with iBHC to the image reconstructed using filtered back-projection (FBP) with water beam hardening correction from computer-simulated noise-free projections. The range of CT numbers has been adjusted in this figure to emphasize this effect; images displayed using the standard window are shown in Figure 3. Figure 2a and b show a clear beam hardening artifact between the highly attenuating Al and PTFE rods. The iBHC option did not improve the accuracy of CT numbers between these two rods. Nevertheless, it notably improved the profile inside the Al rod shown in Figure 4. A comparison of Figure 2a and c shows that the image reconstructed with ADMIRE without iBHC resembled the image reconstructed with FPB; absolute values seen in profiles in Figures 4a and 6a also agreed well. Differences related to smoother transitions at the edges of the PMMA cylinder and the slightly different surroundings of the Al rod for ADMIRE are most likely caused by the Qr36d kernel, which was not used in the FBP. Otherwise, there was a good agreement between the CT numbers produced by both algorithms, see Table 1. The FBP image was produced by DIRA in iteration 0 and so this comparison also demonstrates that DIRA reconstructed realistically looking images.

**Table 1:** CT numbers in ROIs 1–4 (Figure 1)

	Al	PTFE	LDPE	PMMA
80 kV, ADMIRE	2716	980	−108	97
80 kV, ADMIRE, iBHC	2852	976	−108	110
80 kV, DIRA 0th	2724	995	−115	92
Sn150kV, ADMIRE	1685	892	−47	144
Sn150kV, ADMIRE, iBHC	1677	878	−47	147
Sn150kV, DIRA 0th	1705	899	−53	137

### Images produced by Monoenergetic Plus and DIRA

**Figure 5:**
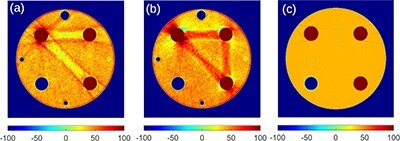
Images computed with Monoenergetic Plus (**a**) without and (**b**) with iBHC and (**c**) DIRA at 40 keV.

**Figure 6:**
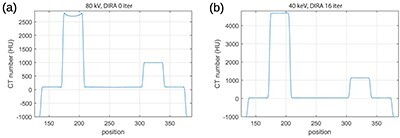
Profiles of images reconstructed with DIRA for (**a**) 0 and (**b**) 16 iterations. The profiles were taken at a horizontal line passing through the aluminium and Teflon rods in Figures 2 and 5.

Virtual monoenergetic images produced by the Monoenergetic Plus algorithm at 40 keV are shown in Figure 5. We recall that these images were derived using Equation (3) from images produced by ADMIRE without and with the iBHC. Again, the range of CT numbers has been adjusted in this figure to emphasize the beam hardening artifact, which mimics the one seen in Figure 2a and b. Figure 5c shows a virtual monoenergetic image at 40 keV produced by DIRA at iteration 16. Note that Monoenergetic Plus without iBHC amplified the beam hardening artefact (see Figures 2a and 5a), while DIRA suppressed it (see Figures 2c and 5c). Also note that Monoenergetic Plus without iBHC amplified the aluminium cupping artefact (see Figure 4a and c), while DIRA suppressed it (see Figure 6a and b).

**Figure 7:**
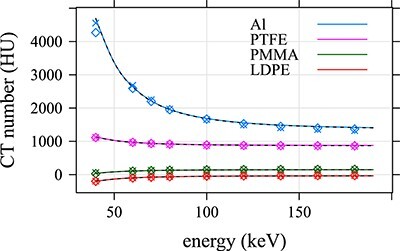
Tabulated CT numbers (black dashed lines) and average CT numbers computed with DIRA (colored solid lines) and Monoenergetic Plus without (colored diamond markers) and with (colored cross markers) iBHC in ROIs 1–4 (Figure 1). Color coding is defined by the figure legend.

**Figure 8:**
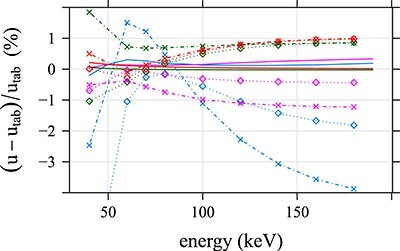
The relative difference between the average LAC in ROIs 1–4 (Figure 1) and tabulated values. The relative difference was calculated as the relative difference of shifted average CT numbers, $u=H+1000$. The relative values are shown for DIRA (Colored solid lines) and Monoenergetic Plus without (Colored diamond markers) and with (colored cross markers) iBHC. The same color coding as in Figure 7 is used. The diamond and cross markers are connected with dotted and dash-dotted lines, respectively, for visual guidance.

Averages of CT numbers produced by both algorithms for the ROIs 1–4 are plotted in Figure 7 and listed in Supplementary Table S1 in the supplementary file available online. The accuracy of these CT numbers was estimated as the relative difference between the determined and tabulated LAC values corresponding to the reported CT numbers. The relative difference between CT numbers is not a good measure since such differences are very high for materials with CT numbers close to 0 HU. The relative difference was calculated as $(\mu -{\mu}_{tab})/{\mu}_{tab}=(u-{u}_{tab})/{u}_{tab}$, where $u=H+1000$ and similarly for the tabulated value ${u}_{tab}$; the relation between $\mu$ and $H$ is given by Equation (1). The relative differences in Figure 8 are larger for Monoenergetic Plus than for DIRA. One of the reasons could be the inaccuracy of the prediction of the (water and iodine) material base calculated from Equation (3); see Supplementary Table S2 in the supplementary file. A similar effect was observed when DIRA used the (water and iodine) base (results are not presented here). Another reason could be an imperfect suppression of the beam hardening artifact by ADMIRE; the iBHC made the values more accurate, but they were not as accurate as the values calculated by DIRA. In the energy range 70–100 keV, both Monoenergetic Plus and DIRA performed well; relative errors were lower than ~1 and 0.4%, respectively. For other energies, however, the relative errors were notably larger for Monoenergetic Plus.

## DISCUSSION

The largest room for improvement of ADMIRE and Monoenergetic Plus seems to be in better suppression of the beam hardening artifact and a choice of the material decomposition doublet.

The use of iBHC improved the situation in the high attenuation objects, but some low-intensity artifacts remained. This may not be a large problem in diagnostic imaging, but for dose calculations in radiotherapy, which requires accurate CT numbers, this behavior is not desirable. The main advantage of ADMIRE compared with FBP is mainly its ability to suppress noise. In DIRA, the suppression is achieved by an iterative reconstruction process that needs ~10 iterations to converge. It increases the reconstruction time. Nevertheless, this time is not critical in the case of radiotherapy treatment planning.

Our observation that the material decomposition to water and iodine in Monoenergetic Plus contributed to the discrepancy between measured and theoretical values at low photon energies is in line with the work of Magnusson *et al.*^(13)^, which shows that the use of this base leads to discrepancies in the 20–40 keV for aluminium. Iodine as a base material is useful for calculating the virtual non-contrast images in angiography using the iodine contrast agent. For radiotherapy, however, other doublets may be more suitable. An in-depth discussion on the parametrization of cross sections is presented by Williamson *et al.*^(14)^. Their recommendation is the (water and calcium chloride solution) doublet or, for low Z materials, the (water and polystyrene) doublet. A review of options available for the two-material decomposition is provided by Heismann *et al.*^(15)^.

In the presented work, a realistic bowtie filter was included in the computer simulation. A comparison with simulations without the bowtie filter (not presented here) showed that the filter was necessary to obtain realistically looking reconstructed images (cf. Figure 2c). A simulation of the bowtie filter is not directly available in DRASIM; the filter was added as a part of the imaged object.

Quantum noise was not simulated to simplify the interpretation of results. Very high noise levels in projection data may lead to a bias in reconstructed CT numbers; nevertheless, previous studies^(9)^ showed that noise levels in the processed measured projection data should result in negligible bias only. Contrary to ADMIRE and Monoenergetic Plus, DIRA does not use any advanced noise suppression techniques. So the inclusion of noise in the simulated data would only hide the observed trends. DIRA is a proof-of-concept code and, as such, has not implemented all the corrections that are used in commercial image reconstruction algorithms.

## CONCLUSIONS

Both the Monoenergetic Plus and DIRA algorithms performed well; the accuracy of attenuation coefficients was better than approximately ±1% at the energy of 70 keV. Compared with DIRA, the worse performance of the Monoenergetic Plus algorithm was caused by its (i) two-material decomposition to iodine and water and (ii) imperfect suppression of the beam hardening artifact.

## FUNDING

This work was supported by Cancerfonden (CAN 2017/1029, CAN 2018/622); grants from the Swedish state under the agreement between the Swedish government and the county councils, the ALF-agreement (LiO-602731); Patientsäkerhetsforskning Region Östergötland [LiO-724181] and Vetenskapsrådet (VR-NT 2016–05033).

## CONFLICT OF INTEREST

The authors declare no conflict of interest with regards to this work.

## Supplementary Material

supplementary_material_ncab108
