# Supplementary material for: ACCURACY OF CT NUMBERS OBTAINED BY DIRA AND MONOENERGETIC PLUS ALGORITHMS IN DUAL-ENERGY COMPUTED TOMOGRAPHY
Source: Radiat Prot Dosimetry. 2021 Jul 16;195(3-4):212–7. doi: 10.1093/rpd/ncab108 (PMC8507448; doi:10.1093/rpd/ncab108)
Supplement: supplementary_material_ncab108 [file supplementary_material_ncab108.pdf]

Supplementary data for the article "Accuracy of CT numbers obtained by DIRA and Monoenergetic Plus algorithms in dual-energy computed tomography"

Maria Magnusson, Michael Sandborg, Gudrun Alm Carlsson, Lilian Henriksson, Åsa Carlsson Tedgren and Alexandr Malusek

Table S1: Average CT number (HU) in regions R1,…,R4 for selected energies E.

MP0: Monoenergetic Plus without iBHC, MP1: Monoenergetic Plus with iBHC, DIR: DIRA, MBP: material base prediction, TAB: tabulated values

	R1: aluminum					R2: Teflon					R3: LDPE					R4: PMMA				
E (keV)	MP0	MP1	DIR	MBP	TAB	MP0	MP1	DIR	MBP	TAB	MP0	MP1	DIR	MBP	TAB	MP0	MP1	DIR	MBP	TAB
40	4268	4554	4682	4464	4694	1114	1118	1130	1112	1129	-202	-198	-200	-190	-202	27	57	38	46	38
60	2591	2684	2640	2676	2629	969	962	973	973	970	-101	-99	-98	-101	-99	103	115	107	106	107
70	2200	2248	2217	2250	2209	935	926	940	940	937	-78	-76	-77	-80	-78	121	129	121	120	121
80	1951	1969	1960	1978	1955	914	903	920	919	917	-63	-62	-64	-66	-65	132	138	130	129	130
100	1669	1654	1687	1673	1684	890	877	899	895	896	-46	-45	-51	-51	-51	145	148	139	139	139
120	1527	1496	1557	1520	1554	878	864	889	883	885	-37	-37	-44	-43	-45	151	153	143	145	143
140	1449	1408	1487	1435	1484	871	857	883	877	878	-32	-32	-41	-39	-41	155	155	146	147	146
160	1402	1356	1446	1384	1443	867	852	880	873	875	-30	-30	-38	-36	-39	157	157	147	149	147
180	1372	1322	1420	1351	1416	864	849	878	870	872	-28	-28	-37	-35	-37	158	158	148	150	148

Table S2: Relative difference (%) between reported and tabulated LAC values in regions R1,…,R4 for selected energies E.

MP0: Monoenergetic Plus without iBHC, MP1: Monoenergetic Plus with iBHC, DIR: DIRA, MBP: material base prediction, TAB: tabulated values

	R1: aluminum				R2: Teflon				R3: LDPE				R4: PMMA			
E (keV)	MP0	MP1	DIR	MBP	MP0	MP1	DIR	MBP	MP0	MP1	DIR	MBP	MP0	MP1	DIR*	MBP
40	-7.48	-2.46	-0.21	-4.04	-0.70	-0.52	0.05	-0.80	0.00	0.50	0.25	1.50	-1.06	1.83	0.00	0.77
60	-1.05	1.52	0.30	1.30	-0.05	-0.41	0.15	0.15	-0.22	0.00	0.11	-0.22	-0.36	0.72	0.00	-0.09
70	-0.28	1.22	0.25	1.28	-0.10	-0.57	0.15	0.15	0.00	0.22	0.11	-0.22	0.00	0.71	0.00	-0.09
80	-0.14	0.47	0.17	0.78	-0.16	-0.73	0.16	0.10	0.21	0.32	0.11	-0.11	0.18	0.71	0.00	-0.09
100	-0.56	-1.12	0.11	-0.41	-0.32	-1.00	0.16	-0.05	0.53	0.63	0.00	0.00	0.53	0.79	0.00	0.00
120	-1.06	-2.27	0.12	-1.33	-0.37	-1.11	0.21	-0.11	0.84	0.84	0.10	0.21	0.70	0.87	0.00	0.17
140	-1.41	-3.06	0.12	-1.97	-0.37	-1.12	0.27	-0.05	0.94	0.94	0.00	0.21	0.79	0.79	0.00	0.09
160	-1.68	-3.56	0.12	-2.42	-0.43	-1.23	0.27	-0.11	0.94	0.94	0.10	0.31	0.87	0.87	0.00	0.17
180	-1.82	-3.89	0.17	-2.69	-0.43	-1.23	0.32	-0.11	0.93	0.93	0.00	0.21	0.87	0.87	0.00	0.17

(*) The relative difference is lower than 0.005%.
